# Body mass index and self-reported body image in German adolescents

**DOI:** 10.1186/s40337-020-00330-3

**Published:** 2020-10-28

**Authors:** Lea Sarrar, Marie Vilalta, Nora Schneider, Christoph U. Correll

**Affiliations:** 1 Department of Psychology, Medical School Berlin, Germany, Rüdesheimer Straße 50, 14197 Berlin, Germany; 2 Department of Audiology and Phoniatrics, Charité University Medicine Berlin, Augustenburger Platz 1, 13353 Berlin, Germany; 3 Department of Child and Adolescent Psychiatry, Psychosomatics and Psychotherapy, Charité University Medicine Berlin, Augustenburger Platz 1, 13353 Berlin, Germany; 4 Department of Psychiatry, The Zucker Hillside Hospital, Northwell Health, 75-59 263rd St, Glen Oaks, NY 11004 USA; 5 Department of Psychiatry and Molecular Medicine, Donald and Barbara Zucker School of Medicine at Hofstra/Northwell, 500 Hofstra Blvd, Hempstead, NY 11549 USA

**Keywords:** Body mass index, Body image, Adolescents, Underweight, Overweight

## Abstract

**Background:**

Despite knowledge about eating disorder symptoms in children and adolescents in the general population, relatively little is known about self-reported and sex-specific eating-disorder-related psychopathology, as well as its specific correlates.

**Methods:**

880 German school-attending adolescents (15.4 ± 2.2 years) and 30 female patients with AN (16.2 ± 1.6 years) were studied. All participants completed the Eating Disorder Inventory-2 and a Body Image Questionnaire.

**Results:**

There were more overweight males than females (15.2% vs 10.1%, *p* < 0.001), but more females with underweight than males (6.2% vs. 2.5%, *p* < .001). Negative body evaluations (*p* < .001) and dissatisfaction (p < .001) were significantly more frequent in females. Compared to underweight female patients with AN, underweight school-attending females had less negative body evaluations (*p* < .001) and lower scores on 5 of the 11 EDI-2 subscales (p < .001; *p* < .05).

**Conclusions:**

Males were more overweight than females, females more underweight. Body image was more important to female than to male youth, yet without reaching pathological values when compared to female patients with AN. Complex emotional and cognitive challenges seem to be a representative factor for eating pathology rather than simply being underweight. These aspects may be relevant for the shift from a thinness-related focus in girls in the general population to the development of an eating disorder.

## Plain English summary

Still too little is known about eating disorder-related psychopathology and its correlates in non-clinical samples, especially with regard to self-report and sex-related differences. Therefore, 880 German school-attending adolescents and 30 female patients with anorexia nervosa (AN) were observed. Males were more overweight than females, females more underweight. Body image was more important to female than to male youth, yet without reaching pathological values. Personality characteristics seem to be maintenance factors in eating disorder pathology, rather than solely being underweight. These aspects may be relevant for the shift from a thinness-related focus in girls in the general population to the development of an eating disorder.

## Background

Disturbed eating behaviours have become a serious concern among adolescents [1]. Severe weight concerns, disordered eating symptoms, and body shape perception disturbances have been reported across cultures [2, 3]. During the last decades a drive for thinness [4] as well as an increasing prevalence of obesity [5] and metabolic syndrome [6] have been observed. Clinically relevant eating disorders are among the most frequent chronic illnesses in adolescents [7]. For the German population, prevalence estimates differ for any threshold eating disorder between 2.9% among females and 0.1% among males, for any subthreshold eating disorder between 2.2% for females and 0.7% for males, and for eating disorder symptoms between 11.5% among females and 1.8% among males [8]. These figures are consistent with those reported in other Western countries [9].

Previous research has demonstrated a high occurrence of disordered eating behaviours in adolescents and suggested the importance of examining preclinical symptoms [10]. Important indicators for eating-disorder-related disturbances are body mass index (BMI; body weight/ body height^2^), body image, eating disorder and related psychopathology, such as clinically relevant perfectionism. In this context, body image refers to the perception of oneself and combines perceptual and cognitive-affective components [11]. A distorted body image is part of the diagnostic criteria of AN [12, 13] and body image dissatisfaction and distortion as well as excessive weight concerns are causally or consequently associated with eating disorders. However, whether body dissatisfaction plays a causal role may vary depending on age and sex [14, 15]. Current findings suggest that body image dissatisfaction is becoming more normative and that sex differences, which implicated females as being more underweight and concerned about body shape and fatness, may be decreasing [2]. Importantly, disordered eating behaviours are associated with an increased risk of further health-compromising behaviours, such as suicide and substance use [16–18]. Thus, the evaluation of non-clinical samples provides an opportunity to observe trends in prevalence and severity of unhealthy BMI status, eating disorders as well as weight and shape concerns, which is important for prevention and treatment programming.

Thus, we aimed to examine BMI, body image, eating-disorder-related psychopathology in a school-attending sample of adolescents and to further compare a subset of the school-attending females who were underweight with a clinical sample of patients with AN. Furthermore, we also aimed to replicate previous results with regard to underweight and overweight among adolescents in Germany. At the same time, we aimed to extend prior findings by applying a multidimensional, including self-reported, assessment battery to males and females, including measurements of body image, eating-disorder-related psychopathology. According to prior findings, we hypothesized a higher occurrence of underweight in females. Furthermore, we assumed less eating-disorder-related psychopathology in the underweight school-attending females compared to female patients with AN.

## Method

### Study population

The study population (Table 1) consisted of 880 school-attending adolescents, of whom 30 school-attending females and 10 school-attending males were underweight (< 10^th^ BMI percentile; see 3.4 below). Therefore, a small and comparable size of sex-matched female patients with AN (*n* = 30) was used for further comparisons with the underweight school-attending females.

**Table 1:** General characteristics of the school-attending and anorexia nervosa sample

Variable	Total school-attending Sample	School-attending females	School-attending males	Female patients with AN	School-attending males vs. School-attending females	School-attending females vs. female patients with AN
		(M ± SD)	(M ± SD)	(M ± SD)	***p***-Value°	
	*n* = 880	*n* = 486	*n* = 394	*n* = 30		
Age		15.4 ± 2.2	15.3 ± 2.2	16.2 ± 1.6	.835	.008**
Height		1.67 ± 0.7	1.76 ± 0.1	1.67 ± 0.1	.665	< .001***
Weight		58.5 ± 10.6	67.9 ± 14.2	42.5 ± 5.9	< .001***	< .001***
BMI		20.9 ± 3.0	21.7 ± 3.2	15.3 ± 1.4	< .001***	< .001***
BMI percent.		54.5 ± 26.6	63.6 ± 24.2	1.3 ± 2.1	< .001***	< .001***

Notes: *n* = number, *M* mean, *SD* standard deviation, *m* meters, *kg* kilogram, °unpaired t-Tests; **p < .01; ***p < .001

Adolescents were recruited through German schools. After having received permission to conduct the study, students were asked to complete the questionnaires during class. The order of assessment administration was varied to avoid sequence effects.

Inclusion criteria for the female patients were meeting the diagnostic criteria of an AN (307.1) according to the Diagnostic and Statistical Manual-V (DSM-V [12]). Exclusion criteria were the presence of another eating disorder according to the DSM-V [12]. The AN diagnosis was confirmed by a structured interview based on DSM (SIAB-EX [19];). Female patients were recruited from the Department for Child and Adolescent Psychiatry, Psychosomatics and Psychotherapy, Charité University Medicine, Berlin.

### Measures

Body height (in meters) and body weight (in kg) were measured with participants wearing lightweight clothing and no shoes by a digital balance scale (manufacturer ‘Korona’, max. 150 kg), and a conventional stadiometer. Subsequently, the BMI (body weight/ body height^2^) and BMI percentiles were calculated as described by Kromeyer-Hauschild et al. [20]. We defined extreme underweight as being ≤ 3^rd^ BMI percentile, underweight as between the > 3^rd^ and ≤ 10^th^ BMI percentile (≤ 17.5 kg/m^2^ for adolescents ≥ 18 years), overweight > 90^th^ to ≤ 97^th^ BMI percentile, and obesity > 97^th^ BMI percentile (more than ≥ 25 to 29.9 kg/m^2^ and ≥ 30 kg/m^2^ for adolescents ≥ 18 years, respectively).

The Body Image Questionnaire [21] is a self-report device assessing clinically relevant body image distortions as well as non-clinical impairments of body image with a two-factor structure [21]. One scale contains items on the negative evaluation of one’s body, the other scale items on the positive perception of one’s body. For this study and the associated research questions, we only included the sum and the mean score of the scale “*Negative Evaluations of the Body*“ as a measure of body image. The internal consistency of the FKB-20 is good (Cronbach’s α = .84) indicating its reliability [21].

The Eating Disorder Inventory-2 [22] is a self-report questionnaire assessing eating-disorder-related psychopathology. It contains 11 scales: *Drive for Thinness* (DT), *Bulimia* (B), *Body Dissatisfaction* (BD), *Ineffectiveness* (I), *Perfectionism* (P), *Interpersonal Distrust* (ID), *Interoceptive Awareness* (IA), *Maturity Fears* (MF), *Asceticism* (A), *Impulse Regulation* (IR), *Social Insecurity* (SI). Internal consistency of the scales is satisfactory (Cronbach’s α = .61-.89, for eating disorder samples, α = .73-.93 [22]).

### Statistical analyses

BMI was calculated for all subjects and categorized into under-, normal- and overweight according to BMI percentiles outlined by Kromeyer-Hauschild et al. [20]. General characteristics of the sample were compared by unpaired t-tests. For the FKB-20, we considered sum scores for the analyses, with regard to EDI-2 age- and sex-adjusted percentiles. FKB-20 and EDI-2 values were divided into quartiles and the groups within these quartiles were compared. Furthermore, for the comparison of underweight girls and patients with AN the subdivision into percentiles was performed. Comparisons of FKB-20 and EDI-2 scores were conducted using Mann-Whitney-U-tests. Additionally, differences between FKB-20 and EDI-2 scores in sex-specific body weight groups were analysed using Kruskal-Wallis-Test. The level of significance was α = 0.05 for all statistical tests that were conducted in SPSS 25. In order to avoid the Type I error, post-hoc Bonferroni corrections (adjusted significance level α = .004) were applied.

## Results

### BMI percentiles and groups

**Fig. 1:**
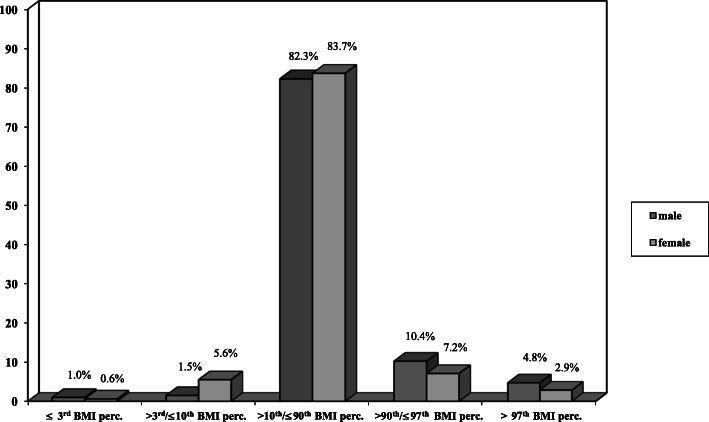
(Extreme) underweight, normal weight, overweight and obesity in adolescents (school-attending sample, *n* = 880)

Altogether, in the school-attending sample, 0.8% were extremely underweight, 3.8% were underweight, 83.0% were normal weight, 8.6% were overweight and 3.8% were obese (Fig. 1). Significantly more females than males (6.2% vs. 2.5%, *p* < .001) were extremely underweight or underweight (≤ 10^th^ BMI percentile), whereas more school-attending males than females (15.2% vs 10.1%, p < .001) were overweight or obese (≥ 90^th^ BMI percentile). All female patients with AN were below the 10^th^ BMI percentile.

### Sex-specific findings on body image and eating-disorder-related psychopathology

Based on Mann-Whitney-U tests, we found significantly higher values among school-attending females for the FKB-20 and on the EDI-2 scales DT, BD and I than in school-attending males. Conversely, school-attending males scored significantly higher on the EDI subscale P (Table 2).

**Table 2:** Sex-specific findings on body image and eating-disorder-related psychopathology

Variable	Quartile	School-attending males	School-attending females	***p***-Value°
FKB-20 *Negative Evaluations of the Body*		*n* = 394	*n* = 486	< .001*
	1.	13.0	15.0	
	2.	16.0	20.0	
	3.	20.0	26.0	
EDI-D		*n* = 223^1^	*n* = 338^1^	< .001*
	1.	0	0	
	2.	0	0	
	3.	0	0.4	
EDI-B	1.	0	0	.414
	2.	0	0	
	3.	0	0	
EDI-BD	1.	0	0	< .001*
	2.	0.1	0.4	
	3.	0. 3	1.1	
EDI-I	1.	0	0	< .001*
	2.	0.1	0.1	
	3.	0.2	0.3	
EDI-P	1.	0.3	0.0	< .001*
	2.	0.5	0.3	
	3.	1.0	0.7	
EDI-ID	1.	0	0	.009
	2.	0.1	0	
	3.	0.4	0.4	
EDI-IA	1.	0	0	.251
	2.	0	0.1	
	3.	0.2	0.2	
EDI-MF	1.	0.3	0.4	.351
	2.	0.5	0.5	
	3.	0.9	0.9	
EDI-A	1.	0	0	.256
	2.	0	0.1	
	3.0	0.3	0.3	
EDI-IR	1.	0	0	.549
	2.	0	0.1	
	3.	0.3	0.3	
EDI-SI	1.	0	0	.440
	2.	0.3	0.3	
	3.	0.5	0.5	

Notes: *n* number, ^1^reduced n due to missing data, *FKB-20* Body Image Questionnaire, *EDI* Eating Disorder Inventory-2, °Mann-Whitney-U-test; *p < .004 (significant group difference after Bonferroni correction)

### Sex-specific findings on body image and eating-disorder-related psychopathology within individual weight percentile groups

Dividing each group according to weight percentiles (extreme underweight/underweight, normal weight and overweight/obese), Kruskal-Wallis-analyses revealed differences between weight percentile groups in school-attending males and in school-attending females for the FKB-20 as well as for the EDI scales, and BD (Table 3).

**Table 3:** Sex-specific findings body image and eating-disorder-related psychopathology within individual weight percentile groups

School-attending males						School-attending females			
Variable		≤ 10^th^ BMI percentile	> 10^th^ to ≤ 90^th^ BMI percentile	> 90^th^ BMI percentile	p-Value°	≤ 10^th^ BMI percentile	> 10^th^ to ≤ 90^th^ BMI percentile	> 90^th^ BMI percentile	p-Value°
	Quartile	n = 10	n = 324	n = 60		n = 30	n = 407	n = 49	
FKB-20 *Negative Evaluations of the Body*	1.	13.3	13.0	15.0	< .001*	14.0	15.0	19.5	< .001*
	2.	15.0	15.0	19.0		18.0	19.0	23.0	
	3.	18.0	19.0	24.0		22.5	26.0	32.0	
EDI-DT		*n* = 5^1^	*n* = 187^1^	*n* = 31^1^	*n* = 223^1^	*n* = 22^1^	*n* = 276^1^	*n* = 40^1^	*n* = 338^1^
	1.	0	0	0	< .001*	0	0	0	< .001*
	2.	0	0	0.1		0	0	0.3	
	3.	0	0	0.4		0	0.4	0.9	
EDI-B	1.	0	0	0	.648	0	0	0	.601
	2.	0	0	0		0	0	0	
	3.	0.1	0	0		0	0	0	
EDI-BD	1.	0	0	0.3	< .001*	0	0	0.4	< .001*
	2.	0	0	0. 8		0.2	0.4	1.1	
	3.	0. 6	0.3	1.3		0.6	1.0	2.0	
EDI-I	1.	0	0	0	.264	0	0	0	.414
	2.	0.2	0	0.1		0.2	0.1	0.2	
	3.	0.5	0.2	0.3		0.3	0.3	0.4	
EDI-P	1.	0.5	0.3	0.2	.828	0	0	0	.661
	2.	0.5	0.7	0.3		0.3	0.3	0.3	
	3.	1.7	1.0	0.5		0.6	0.7	0.7	
EDI-ID	1.	0.1	0.3	0.2	.373	0	0	0	.330
	2.	0.4	0.7	0.3		0	0	0	
	3.	0.8	1.0	0.5		0.1	0.3	0.3	
EDI-IA	1.	0	0	0	.102	0	0	0	.410
	2.	0	0	0.1		0	0.1	0.1	
	3.	0.1	0.2	0.3		0.2	0.2	0.3	
EDI-MF	1.	0.3	0.3	0.4	.380	0.2	0.4	0.3	.218
	2.	0.6	0.5	0.5		0.4	0.5	0.5	
	3.	1.6	0.8	1.0		0.8	0.9	0.8	
EDI-A	1.	0	0	0	.112	0	0	0	.010
	2.	0	0	0.1		0	0.1	0.3	
	3.	0.3	0.3	0.4		0.1	0.3	0.5	
EDI-IR	1.	0	0	0	.818	0	0	0	.546
	2.	0	0	0.1		0.1	0	0.1	
	3.	0.6	0.3	0.2		0.3	0.3	0.3	
EDI-SI	1.	0.1	0	0.1	.621	0	0	0	.578
	2.	0.3	0.3	0.3		0.4	0.3	0.3	
	3.	0.9	0.5	0.5		0.5	0.5	0.7	

Notes: *n* number, ^1^reduced n due to missing data, *FKB-20* Body Image Questionnaire, *EDI* Eating Disorder Inventory-2, °Kruskal-Wallis; *p < .004 (significant group difference after Bonferroni correction)

### Comparison of underweight school-attending males and females and school-attending females with female patients with AN

Based on the weight percentile groups, we compared the school-attending males (*n* = 10) and females (*n* = 30) who were underweight (≤ 10^th^ percentile) as well as female patients with AN (*n* = 30). Pairwise comparisons for males vs. females who were underweight (Mann-Whitney-U) revealed no significant group differences. Comparing female patients with AN to school-attending females with underweight (Table 4), we found significantly higher scores for the AN group regarding the FKB-20 as well as on the following EDI-2 subscales: DT, BD, IA, A, and ID.

**Table 4:** Differences between school-attending females with underweight and female patients with AN

Variable	Percentile	School-attending females ≤ 10^th^ BMI percentile	Female patients with AN ≤ 10th BMI percentile	p-Value°
		*n* = 30	*n* = 29^1^	< .001*
FKB-20 *Negative Evaluations of the Body*	25.	14.0	24.5	
	50.	18.0	34.0	
	75.	22.5	40.5	
		*n* = 22^1^	*n* = 30	< .001*
EDI-DT	25.	0	0.4	
	50.	0	1.1	
	75.	0	2.1	
EDI-B	25.	0	0	.151
	50.	0	0	
	75.	0	0.3	
EDI-BD	25.	0	0.8	< .001*
	50.	0.2	1.2	
	75.	0.6	1.6	
EDI-I	25.	0	0.1	.005
	50.	0.2	0.7	
	75.	0.3	1.1	
EDI-P	25.	0	0.3	.030
	50.	0.3	0.7	
	75.	0.6	1.0	
EDI-ID	25.	0	0	.004*
	50.	0	0.1	
	75.	0.1	0.6	
EDI-IA	25.	0	0.1	< .001*
	50.	0	0.4	
	75.	0.2	0.6	
EDI-MF	25.	0.2	0.4	.202
	50.	0.4	0.5	
	75.	0.8	0.9	
EDI-A	25.	0	0.3	< .001*
	50.	0	0.4	
	75.	0.1	0.6	
EDI-IR	25.	0	0	.698
	50.	0.1	0.1	
	75.	0.3	0.3	
EDI-SI	25.	0	0.1	.035
	50.	0.4	0.6	
	75.	0.5	1.1	

Notes: *n* number, ^1^reduced n due to missing data, *FKB-20* Body Image Questionnaire, *EDI* Eating Disorder Inventory-2, *AN* anorexia nervosa, °Mann-Whitney-U-test; *p < .004 (significant group difference after Bonferroni correction)

## Discussion

### BMI, body image and eating-disorder-related psychopathology

Our findings revealed that 15.2% of male and 10.1% of female adolescents in our school-attending study population were *overweight or obese*. These data are comparable with data by a recent German study as well as European and international studies [5, 23–25]. Although there is some evidence that the rise in the prevalence of overweight and obesity is plateauing [5, 26, 27], prevalence rates are still high and general shifts in the BMI-distribution were found during the last decades [27]. The National Health and Nutrition Examination Survey reported a significantly increasing linear trend in obesity between 1999 and 2000 and 2015-2016, in both adults and youth in the US [28].

*Underweight* status was more prevalent among school-attending females (6.2%) than school-attending males (2.5%) and occurred less often than overweight/obesity in our study. Accordingly, Schienkiewitz et al. [29] observed comparable rates of underweight in children and adolescents, although no sex differences were found. Nevertheless, other studies have shown sex differences with more underweight in females than males (for example Grajda et al. [30]). Sex differences regarding over- and underweight may result from underlying sociocultural and psychological differences. For instance, males and females differ in calorie consumption, eating styles [31] and body fat distribution [32]. Additionally, females experience more body weight and thinness-oriented body dissatisfaction than males [2, 33].

Our finding regarding more prevalent negative *body evaluations and body dissatisfaction* in school-attending females than school-attending males contrasts with other studies that reported an equal distribution of body dissatisfaction among both boys and girls [2, 34, 35]. However, these studies also report a significantly more thinness-oriented dissatisfaction among females, but also a more muscle-oriented dissatisfaction among males [2, 34, 35].

Regarding *eating-disorder-related psychopathology* we found more ineffectiveness in school-attending females compared with school-attending males which is in line with other findings [36] and could be related to negative body evaluations and body dissatisfaction in the school-attending females in the present study. Surprisingly there was more perfectionism in the school-attending males. It is possible that this finding may also be associated with body-related dissatisfaction in males, but as reported, this finding seems to be more related to a (male-specific) kind of body ideal and dissatisfaction in terms of drive for muscularity than thinness [2, 34, 35]. However, these specific aspects cannot be operationalised with the commonly utilized measures that were used in this study.

Results regarding *body image and eating-disorder-related psychopathology within* the groups of males and females and *individual weight percentile groups* demonstrated that significant differences were found only on body image-related scales. Both sexes showed more body image dissatisfaction in the presence of underweight than in the presence of normal or overweight. This finding is consistent with other studies [37] and underlines that body image dissatisfaction and eating disorder psychopathology are strongly linked, even in school-attending samples.

A *comparison of the underweight school-attending females and female patients with AN* showed greater body image dissatisfaction for the latter. In addition, female patients with AN showed significantly higher scores on the EDI-2 scales of interpersonal distrust, interoceptive awareness and asceticism, pointing out complex emotional and cognitive challenges. The increased values on the eating-disorder-related scales could indicate that these aspects are specific to the eating disorder psychopathology, rather than the underweight status itself. In order to assess eating-disorder-related psychopathology among young people in the general population, the underlying factors mentioned above should be focused on in addition to exclusively screening for drive for thinness [38, 39].

### Strengths and limitations

When interpreting the results of this study, certain limitations have to be taken into account. First, we used self-report questionnaires. Methodologically, the additional use of a structured interview in our school-attending sample would have been advantageous. Nevertheless, the fact of testing during school days and the related lack of time did not allow for time-consuming individual interviews in such a large sample. For the same reason, we were not able to diagnose any serious eating disorders according to the DSM-V [12] in our school-attending sample. Therefore, we cannot eliminate the possibility of clinically relevant eating disorders in the underweight subgroup and it could be false negatives in the school-attending sample. In addition, underweight school-attending females and males in this study may have eating disorder psychopathology with a different severity or duration than those of patients with AN. Due to different and partly small age subgroup sizes in the school-attending survey sample, we have not carried out analyses within these groups, so that the results regarding under- and overweight as well as obesity only apply to the entire sample. As we only screened for underweight and overweight/obesity and the only disease control group was represented by female patients with AN, we cannot generalize our data to other forms of disordered eating behaviour, such as bulimic or binge eating symptoms. Furthermore, we did not collect information about the subtype of AN (restrictive/binge-purge). Moreover, as we did not collect information about the sociodemographic information of all attendees of the schools from which our school-attending sample was drawn, the degree of representativeness of the sample is unclear. Nevertheless, the sex distribution and body measures in our school-attending sample did match well with the previously reported epidemiological data in Germany [40, 41]. In addition, the small size of the patients with AN as well as underweight school-attending females and males limits the representativeness of these samples and generalization of the related findings. Moreover, no control group with AN was available for the underweight school-attending males of our sample. Due to the sample size compared to studies with an epidemiological approach, findings of the present study regarding over- and underweight as well as obese have to be interpreted very carefully. In addition, our data must be treated with caution, as the BMI percentile calculations are based on a reference group from data sets from between 1985 and 1999 [20]. A shift in these percentiles seems possible. Besides, because of the cross-sectional nature of our study we are not able to evaluate if underweight, body image dissatisfaction or other factors could be predictors of eating disorder symptoms.

Despite these limitations, strengths of this study include its relatively large sample size of the school-attending cohort, the multidimensional assessment of body image, eating-disorder-related psychopathology, body height and body weight, and the control group of female patients with AN.

## Conclusion

We observed sex differences in the prevalence of (extreme) underweight and overweight/obese in a German school-attending sample. Body image concerns were more prevalent among school-attending females than males. Underweight by itself does not seem to be a representative factor for eating pathology, as female patients with AN differed significantly from school-attending females with underweight in psychopathological factors. These findings underline the importance of a multidimensional assessment of body image and eating-disorder-related psychopathology including self-reports when characterizing an underweight as well as potentially eating disordered sample. Therefore, the evaluation of these aspects in non-clinical samples is important to detect current prevalence rates, and trends in behaviours and attitudes. Further studies should focus on these issues instead of exclusively screening for BMI in non-clinical samples as well as patients with AN. Preventive and treatment programmes should be based on knowledge of underweight and dissatisfaction with body image, but should also focus on emotional, cognitive and personal temperament factors who may be involved in the development of eating disorders. Psychotherapeutic approaches in the treatment of AN and measurement of psychotherapy success should urgently focus these factors in addition to focusing in the key outcome of weight gain and weight restoration. For all, preventive and treatment programmes as well as psychotherapeutic approaches it could be helpful to take personality functions into account as they are described by the Operationalized Psychodynamic Diagnostic System [42] and associated with a lot of psychological disorders in childhood and adolescence including eating disorders [43, 44]. Regarding body dissatisfaction there are indications for a significantly more muscularity-related body ideal in boys [2, 34, 35], which should be deepening investigated in future studies. Existing test instruments should be adapted to these findings.

## Data Availability

Due to the nature of this research, participants of this study did not agree for their data to be shared publicly, so supporting data is not available.

## Abbreviations

A: Asceticism
AN: Anorexia nervosa
B: Bulimia
BD: Body Dissatisfaction
BMI: Body Mass Index
DSM: Diagnostic and Statistical Manual
DT: Drive for Thinness
EDI-2: Eating Disorder Inventory-2
FKB-20: Body image questionnaire
I: Ineffectiveness
IA: Interoceptive Awareness
ID: Interpersonal Distrust
IR: Impulse Regulation
MF: Maturity Fears
P: Perfectionism
SI: Social Insecurity
